# Structural isomserism in gold nanoparticles revealed by X-ray crystallography

**DOI:** 10.1038/ncomms9667

**Published:** 2015-10-20

**Authors:** Shubo Tian, Yi-Zhi Li, Man-Bo Li, Jinyun Yuan, Jinlong Yang, Zhikun Wu, Rongchao Jin

**Affiliations:** 1 Key Laboratory of Materials Physics, Anhui Key Laboratory of Nanomaterials and Nanotechnology, Institute of Solid State Physics, Chinese Academy of Sciences, Hefei, Anhui 230031, China; 2 State Key Laboratory of Coordination Chemistry, School of Chemistry and Chemical Engineering, Nanjing University, Nanjing 210093, China; 3 Hefei National Laboratory for Physical Sciences at the Microscale and Synergetic Innovation Center of Quantum Information and Quantum Physics, University of Science and Technology of China, Hefei, Anhui 230026, China; 4 Department of Chemistry, Carnegie Mellon University, Pittsburgh, Pennsylvania 15213, United States

## Abstract

Revealing structural isomerism in nanoparticles using single-crystal X-ray crystallography remains a largely unresolved task, although it has been theoretically predicted with some experimental clues. Here we report a pair of structural isomers, Au_38T_ and Au_38Q_, as evidenced using electrospray ionization mass spectrometry, X-ray photoelectron spectroscopy, thermogravimetric analysis and indisputable single-crystal X-ray crystallography. The two isomers show different optical and catalytic properties, and differences in stability. In addition, the less stable Au_38T_ can be irreversibly transformed to the more stable Au_38Q_ at 50 °C in toluene. This work may represent an important advance in revealing structural isomerism at the nanoscale.

## Abstract (web summary)

Revealing structural isomerism in nanoparticles remains a largely unresolved task. Here, the authors use several techniques, including single-crystal X-ray crystallography, to characterize two structural isomers of Au_38_, and report their different optical and catalytic properties and differences in stability.

## Graphical abstract

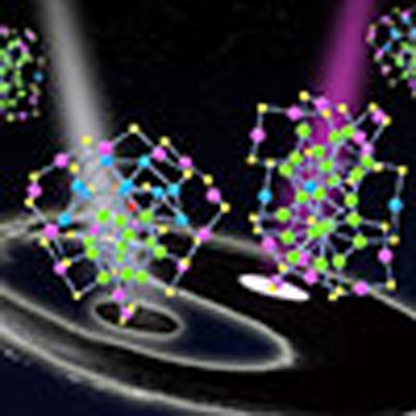

Structural isomerism in organic molecules is a common occurrence due to the bonding diversity of carbon. However, for nanoscale or even larger scale materials, experimental observation of structural isomerism has been largely impeded by the challenge of unravelling the intrinsic structure at the atomic level^1^. Nevertheless, theoretical and experimental efforts^2^,^3^,^4^,^5^,^6^,^7^,^8^ in searching for structural isomerism in such materials continue, because such a finding would provide precise and insightful structure–property correlations and meaningful guidance for designing and synthesizing unique functional materials. The recently developed ultrasmall, thiolated metal nanoparticles (also called nanoclusters) provide opportunities for investigating structural isomerism, as they can now be controlled with atomic precision^9^,^10^,^11^,^12^,^13^,^14^,^15^,^16^,^17^,^18^,^19^,^20^ and their structures can be resolved by single-crystal X-ray crystallography (SCXC) as well. To date, the structures of a series of thiolated metal nanoparticles with various sizes have been elucidated experimentally and theoretically^21^,^22^,^23^,^24^,^25^,^26^,^27^,^28^; however, to the best of our knowledge, no structural isomerism in thiolated metal nanoparticles has been reported, albeit Au_24_(SCH_2_Ph-^t^Bu)_20_ and Au_24_(SePh)_20_ were revealed to have different Au_24_ core structures^29^,^30^. In a strict sense, Au_24_(SCH_2_Ph-^t^Bu)_20_ and Au_24_(SePh)_20_ are not structural isomers, since their ligands are different. Thus, structural isomerism in thiolated nanoparticles remains a mystery.

In the current work, using a modified synthesis method for Au_25_, we synthesize a nanocluster, whose composition is determined to be the same as that of the previously reported Au_38_(PET)_24_ (refs 31, 32, 33) (PET, phenylethanethiolate), as evidenced by electrospray ionization mass spectrometry (ESI–MS) in combination with X-ray photoelectron spectroscopy (XPS) and thermogravimetric analysis (TGA). SCXC reveals that the structure of this nanocluster is different from that of the previously reported structure^24^. To differentiate the two structures, the previous Au_38_ is denoted as Au_38Q_ and our nanocluster is denoted as Au_38T_ (where Q and T are the surname initial of the first author of the previous and current work, respectively). Au_38T_ and Au_38Q_ are therefore structural isomers and they represent the first pair of structural isomers in nanoparticles as revealed by SCXC, to the best of our knowledge. The two isomers exhibit distinctly different optical, stability and catalytic properties, and the less stable Au_38T_ can be irreversibly transformed to the more stable Au_38Q_ at 50 °C.

## Results

### Characterization

**Figure 1:**
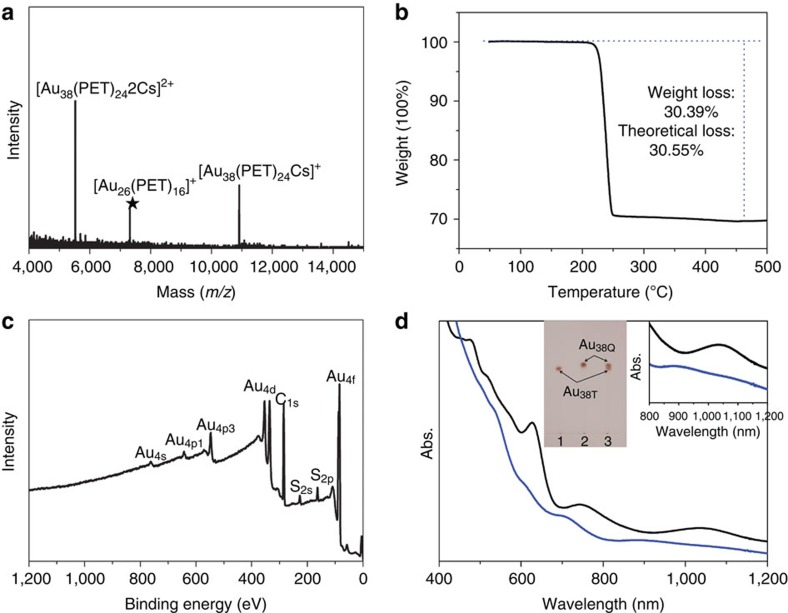
Characteriztion of Au_38T_. (**a**) ESI mass spectrum of the Au_38T_. (**b**) TGA of Au_38T_. (**c**) XPS spectrum of Au_38T_. (**d**) Ultraviolet–visible–near-infrared absorption spectra of Au_38T_ (blue) and Au_38Q_ (black) in toluene (measurement temperature: 0 °C). Insets are the photo of thin-layer chromatography, and enlarged absorption spectra in the range from 800 to 1,200 nm of Au_38T_ and Au_38Q_.

Au_38T_ was synthesized using a modified one-pot method^34^ and isolated using preparative thin-layer chromatography (PTLC)^35^,^36^. ESI–MS was employed to determine the exact molecular mass and formula of the novel nanoparticle (note: caesium acetate was added to form positively charged adducts). Three distinct peaks centred at *m/z* 10910.186, 7317.312 and 5522.217 were observed in the mass spectrum (Fig. 1a). The peaks at *m/z* 10910.186 and 5522.217 (almost half of 10910.186) can be readily assigned to [Au_38_(PET)_24_Cs]^+^ (theoretical *m/z* value: 10910.658; deviation: 0.472) and [Au_38_(PET)_24_Cs_2_]^2+^ (theoretical *m/z* value: 5522.772; deviation: 0.555), respectively. The peak at m/z 7317.312 can be assigned to Au_26_(PET)_16_ (theoretical *m/z* value: 7316.818; deviation: 0.494), which could be a fragment of Au_38_(PET)_24_, because the nanoparticles are monodisperse, as demonstrated by TLC, and it is also observed in the ESI spectrum of Au_38Q_ (see below). Based on the ESI–MS results, it is concluded that the as-prepared nanoparticle is neutral, and that its composition is Au_38_(PET)_24_, which is also corroborated by the TGA and XPS. TGA shows a weight loss of 30.39 wt% (Fig. 1b), corresponding to the theoretical loss of 30.55 wt% according to the formula. No other elements (including Cl, Br, N and Na) was detected by XPS (Fig. 1c), which excludes the possibility of existence of potential counterions such as Cl^−^, Br^−^, [N(C_8_H_17_)_4_]^+^ and Na^+^; thus, the as-prepared nanoparticle is neutral. Quantitative measurement reveals that the Au/S atomic ratio is 38.0:24.3 (Supplementary Figs 3 and 4), in good agreement with the expected ratio (38.0:24.0) for the composition of Au_38_(PET)_24_. Thus, the formula is identical to that of the nanoparticle previously reported in ref. 31; however, the absorption spectrum of our nanoparticle distinctly differs from that of the previous nanoparticle. The ultraviolet–visible–near-infrared spectrum of the novel Au_38_(PET)_24_ (abbreviated as Au_38T_) shows six absorption peaks at 505 nm (ɛ: 3.86 gcm l^−1^), 540 nm (ɛ: 3.22 gcm l^−1^), 610 nm (ɛ: 1.46 gcm l^−1^), 700 nm (ɛ: 0.69 gcm l^−1^), 880 nm and 1,090 nm (Fig. 1d and Supplementary Fig. 2). The previous Au_38_(PET)_24_ (abbreviated as Au_38Q_) shows six absorption peaks centred at 480 nm (ɛ: 4.62 gcm l^−1^), 520 nm (ɛ: 3.72 gcm l^−1^), 570 nm (ɛ: 2.86 gcm l^−1^), 627 nm (ɛ: 2.59 gcm l^−1^), 740 nm (ɛ: 0.58 gcm l^−1^) and 1,035 nm (Fig. 1d and Supplementary Fig.1). TLC also indicates that they are not the same nanoparticle (Fig. 1d, inset). Indeed, they are a pair of structural isomers (vide infra).

### Atomic structure

**Figure 2:**
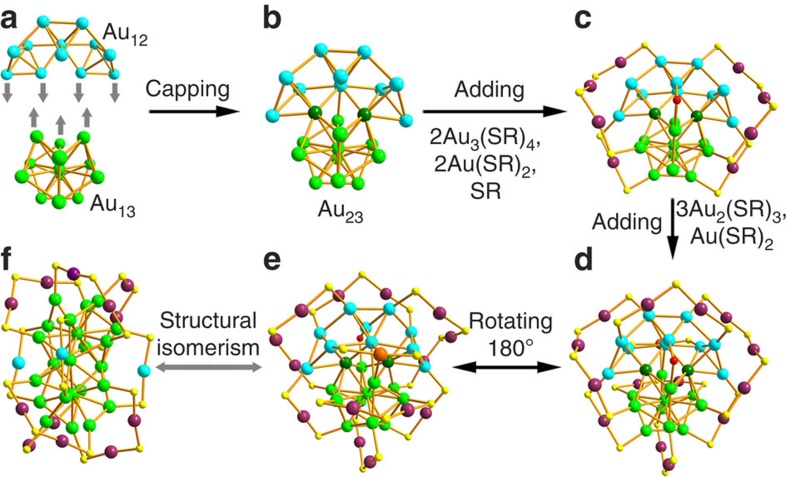
Structures of Au_38T_ and Au_38Q_. (**a**) Anatomy of the Au_23_ core, which consists of a Au_12_ cap unit and Au_13_ icosahedral unit. (**b**) Au_23_ core, which is constructed by one Au_12_ unit and one Au_13_ unit sharing two gold atoms. (**c**) Two Au_3_(SR)_4_, two Au(SR)_2_ and one SR linking the Au_12_ cap and Au_13_ icosahedron. (**d**) Three Au_2_(SR)_3_ and one Au(SR)_2_ protecting the Au_23_ core. (**e**) Back view of Au_38T_. (**f**) The Au_38Q_ structure.

**Figure 3:**
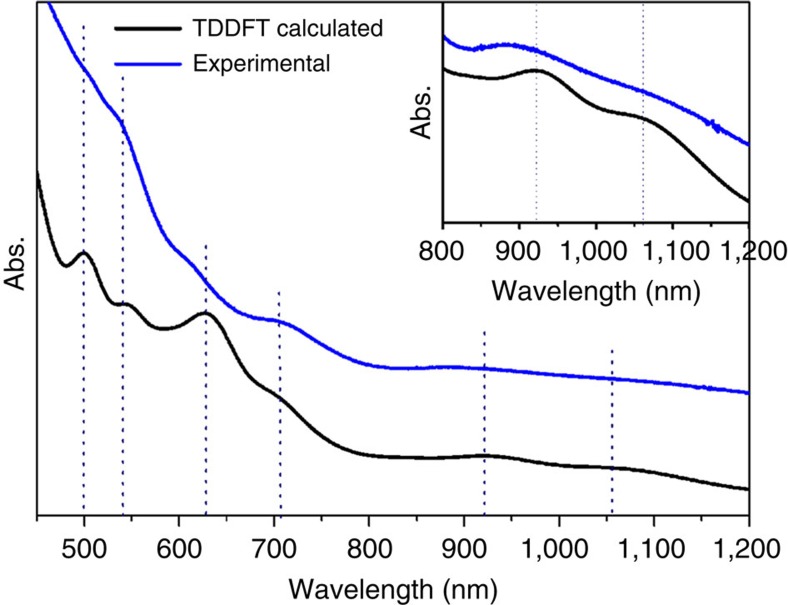
Comparison of ultraviolet–visible–near-infrared absorption spectra of Au_38T_. Blue: experimental; black: calculated by time-dependent density function (TDDFT) method. Inset is the enlarged spectra in the range from 800 to 1,200 nm.

The structure of the previous Au_38Q_ was determined by SCXC and it has a core-shell structure consisting of a face-fused bi-icosahedral Au_23_ core, which is capped by a second shell composed of the remaining 15 gold atoms (Fig. 2f). To confirm that our nanoparticle (Au_38T_) is an isomer of Au_38Q_, we grew high-quality single crystals and successfully elucidated the structure via SCXC. Briefly, the new structure of Au_38T_ is composed of one Au_23_ core and one mixed capping layer of thiolate ligands and gold–thiolate complex units. The Au_23_ core consists of one icosahedral Au_13_ and one Au_10_ unit, and the mixed surface layer contains two Au_3_(SR)_4_ staple units, three Au_2_(SR)_3_ staple units, three Au_1_(SR)_2_ staple units and one bridging thiolate SR ligand. The anatomy of the Au_38T_ structure starts with the central Au_23_ core (Fig. 2b), which can be viewed as one Au_12_ cap and one Au_13_ icosahedron (Fig. 2a) fused together via sharing two gold atoms (Fig. 2b, dark green gold atoms), which is in distinct contrast with the case of Au_38Q_; for the latter, the two Au_13_ icosahedra are fused together via sharing a face (three gold atoms) to form a bi-icosahedral Au_23_ core. The Au_12_ cap is composed of three tetrahedra and the Au–Au bond lengths in each tetrahedron range from 2.71 to 2.88 Å. In the Au_13_ icosahedron, the Au–Au bond lengths between the central atom and the shell Au atoms (except for the two shared gold atoms) vary from 2.71 to 2.82 Å. The bond lengths between the two shared gold atoms and the central atom of Au_13_ icosahedron are 2.77 and 2.78 Å, respectively. The different Au_23_ core in Au_38T_ (in contrast to the biicosahedral Au_23_ core of Au_38Q_) leads to various surface-binding structures. The Au_23_ core in our case was capped by two Au_3_(SR)_4_ units and two Au(SR)_2_ units, and the average Au–S bond lengths/Au–S–Au bond angles were 2.33 Å/96.47° and 2.32 Å/94.43° in the Au_3_(SR)_4_ and Au(SR)_2_ staple units, respectively (Fig. 2c). Interestingly, in addition to the two Au_3_(SR)_4_ units and two Au(SR)_2_ units, one bridging thiolate (SR) is also found to link the Au_13_ icosahedron and the Au_12_ cap (Fig. 2c, the sulfur atom is marked in red), the two Au–S bond lengths are 2.33 and 2.30 Å, respectively, and the Au–S–Au bond angle is 92.66°. It is noteworthy that in Au_38Q_, the Au_23_ core was protected by six Au_2_(SR)_3_ and three Au(SR)_2_ staple units; no comparable Au_3_(SR)_4_ staple units and bridging thiolate (SR) was observed. The Au_13_ icosahedron in Au_38T_ is exclusively capped by two Au_2_(SR)_3_ staple units (the average Au–S bond lengths in the Au_2_(SR)_3_ staple units are 2.35 and 2.34 Å, respectively, and the average Au–S–Au bond angles in the Au_2_(SR)_3_ staple units are 92.20° and 90.33°, respectively) and the Au_12_ cap is capped by one Au_2_(SR)_3_ staple unit (the average Au–S bond length is 2.33 Å and the average Au–S–Au bond angle is 97.03°). In addition, the Au_12_ cap is also capped by one Au(SR)_2_ staple unit, and the average Au–S bond length/Au–S–Au bond angle are 2.33 Å and 99.91° (Fig. 2d). The structure resolved by X-ray diffraction was further analysed by computations: the simulated ultraviolet–visible–near-infrared spectrum is close to the experimental one (Fig. 3).

As discussed above, the structure of Au_38T_ is remarkably different from that of Au_38Q_ and the main differences between the two structures lie in the type of Au_23_ core and the surface capping mode of the Au_23_ core. Au_38T_ and Au_38Q_ have an identical composition but completely different structures; thus, they are literally a pair of structural isomers. Notably, the structure of Au_38T_ reported in this work is novel and also differs from those theoretical structures predicted by Hakkinen *et al*.^37^, Tsukuda and colleagues^38^, Jiang *et al*.^39^ and Zeng and colleagues^40^, among others.

### Transformation

**Figure 4:**
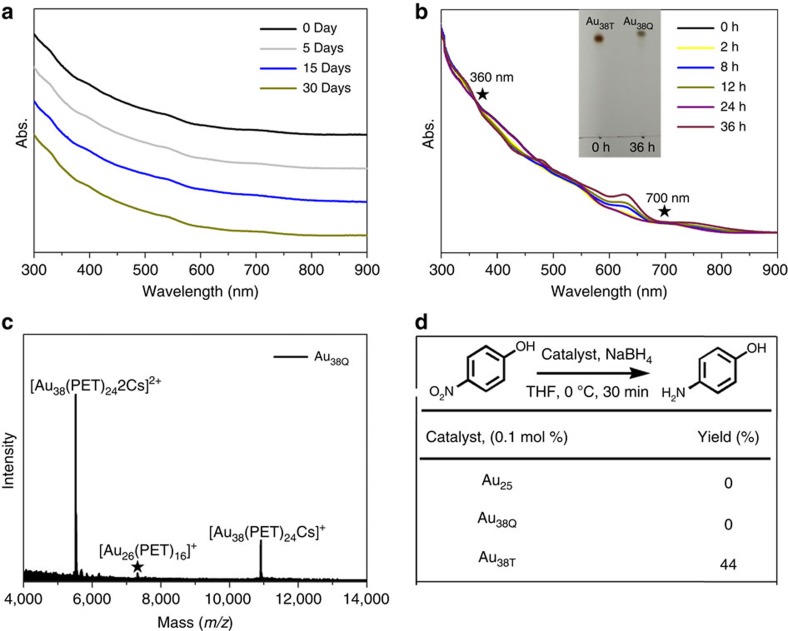
Difference in stability and catalysis between Au_38T_ and Au_38Q_. (**a**) Time-dependent ultraviolet–visible–near-infrared absorption spectra of Au_38T_ at −10 °C in toluene. (**b**) Ultraviolet–visible–near-infrared absorption spectral transformation at 50 °C in toluene (the isosbestic points are at 360 and 700 nm). Inset: thin-layer chromatography of Au_38T_ before and after the transformation. (**c**) ESI mass spectrum of the transformed product. (**d**) Catalytic activities of Au_25_, Au_38T_ and Au_38Q_.

Au_38T_ exhibits relatively high stability at low temperatures, as no obvious spectral changes was detected when a solution of Au_38T_ was stored at −10 °C for as long as 1 month in toluene (Fig. 4a). However, the absorption spectrum of Au_38T_ gradually changed to that of Au_38Q_ at 50 °C in toluene (Fig. 4b), which indicates that Au_38T_ can transform to Au_38Q_ at elevated temperatures. TLC and ESI–MS further support this transformation (Fig. 4b (inset) and Fig. 4c). However, the reverse transformation (that is, from Au_38Q_ to Au_38T_) was not successful under various investigated conditions. These results indicate that Au_38T_ is less stable than Au_38Q_, and that Au_38T_ can only be irreversibly transformed to Au_38Q_. The reason for why the relatively unstable Au_38T_ is formed rather than the stable Au_38Q_ during the synthesis is probably because the former is kinetically favourable in our reaction conditions, similar to some previous reports^41^,^42^.

### Catalysis

Au_38T_ exhibits remarkably higher catalytic activity than Au_38Q_ at low temperature (for example, 0 °C) in reduction reactions. For example, 4-nitrophenol can be reduced to 4-aminophenol in 44% yield with 0.1 mol% Au_38T_ catalyst in half an hour, whereas no reduction occurred when Au_25_(PET)_18_^−^TOA^+^ (Au_25_ for short, TOA^+^: tetra-*n*-octylammonium) or Au_38Q_ was used as the catalyst (Fig. 4d and Supplementary Fig. 5) under the same reaction conditions. It is noteworthy that in other cases, Au_25_ was reported to exhibit good catalytic reduction activity^43^,^44^. The high catalytic activity of Au_38T_ may be due to its surface being not as densely protected as the surfaces of Au_25_ and Au_38Q_; further investigation is underway. A previous work^44^ implied that the catalytic properties of gold nanoclusters are not only size dependent but also structure sensitive. However, the structure dependence of catalytic properties was unclear at that time, because the ligands were different in Au_44_(PET)_32_ and Au_44_(TBBT)_28_ (TBBT: 4-tert-butylbenzenethiolate), and the ligand effect should be considered. Herein, it is unambiguously demonstrated that the structure effect indeed exists, because Au_38T_ and Au_38Q_ exhibit remarkably different catalytic performance. Au_38T_ is relatively robust and can retain its ultraviolet–visible–near-infrared spectrum even after 18 catalytic cycles (Supplementary Fig. 6). It is noteworthy that the gradual decrease in the yield is primarily due to the unavoidable mass loss of the catalyst during the isolation by column chromatography. However, after 21 cycles, the catalyst transformed to more stable Au_38Q_ demonstrated by the ultraviolet–visible–near-infrared spectra and accordingly the loss of catalytic activity (see Supplementary Table 1). The high catalytic activity at low temperatures indicates the potential application of Au_38T_ in some catalytic processes.

## Discussion

In summary, we have discovered a pair of structural isomers Au_38T_ and Au_38Q_, which were identified using ESI–MS, TGA, XPS and SCXC. Although both species have the same composition (that is, Au_38_(PET)_24_), they have distinctly different structures, which results in differences in their optical and catalytic properties, as well as structural stability. The less stable Au_38T_ can be irreversibly transformed to the more stable Au_38Q_ at high temperatures. The structure of Au_38T_ is very interesting: it is composed of a Au_23_ core (fused by one Au_13_ icosahedron and one Au_12_ cap by sharing two atoms) and a mixed layer of thiolate ligands and gold–thiolate complex units for surface protection. This structure is unique (that is, not found in other reported gold nanoclusters). In particular, the diversity of staple units and the bridging thiolate found in Au_38T_ provide a new direction for structural studies of metal nanoclusters. The significance and novelty of this work are as follows. (i) A novel synthesis method is developed, with which a novel gold nanoparticle is readily synthesized, and the composition of the as-prepared nanoparticle is precisely determined using ESI–MS in conjunction with XPS and TGA. (ii) The structure of Au_38T_ is resolved using SCXC and the unique structural features provide important implications for nanocluster structural studies. (iii) Significantly, structural isomerism is observed in nanoparticles for the first time. (iv) The distinctly different properties (in particular the catalytic properties) of the two structural isomers indicate a structure–property correlation and this will have important implications for future catalytic studies. It is expected that our work may motivate more studies on structural isomerism and structure–property correlations in nanoscale or even larger scale materials.

## Methods

### Reagents

All chemicals and reagents are commercially available and were used as received. Tetraoctylammonium bromide (TOAB, 98.0%) and 4-nitrophenol (99.0%) were obtained from Aladdin; 2-phenylethanethiol (PhC_2_H_4_SH, 99.0%) was purchased from Sigma-Aldrich; Au_38Q_ and Au_25_(PET)_18_^−^TOA^+^ were synthesized following reported methods^31^,^34^.

### Synthesis

Au_38T_ was synthesized using a modified one-pot method and separated using PTLC. Briefly, HAuCl_4_·4H_2_O (0.20 g, 0.48 mmol) was mixed with 1.03 equivalents of TOAB (0.27 g, 0.49 mmol) in CH_2_Cl_2_ (40 ml). Then, 9 equivalents of phenylethanethiol (0.61 ml, 4.50 mmol) were added to this solution and the solution was stirred for ∼2 h until it became colourless. To this solution, 5.3 equivalents of NaBH_4_ (0.11 g, 2.80 mmol) in cold water (5 ml) was added in one shot under vigorous stirring and the reaction was allowed to proceed under constant stirring for 6 h. CH_2_Cl_2_ was removed via rotary evaporation at 20 °C to isolate the crude product. For purification, the crude product was extracted with a small amount of tetrahydrofuran (THF) and washed with ice water three times and with CH_3_OH two times; during this procedure, traces of inorganic salt, excess TOAB and phenylethanethiol were thoroughly removed. Next, the as-obtained crude products were separated using PTLC (dichloromethane: petroleum ether=3:4) and finally the target product was isolated from the reddish brown band of PTLC after extraction with CH_2_Cl_2_, with a yield of 5%.

### Characterization

The ultraviolet–visible–near-infrared absorption spectrum was measured on a UV-3600 spectrophotometer (Shimadzu, Japan) at room temperature. TGA analysis was conducted under a N_2_ atmosphere (∼3 mg sample used, flow rate ∼50 ml min^−1^) on a TG/DTA 6300 analyzer (Seiko Instruments, Inc.) and the heating rate was 10 °C min^−1^. XPS measurements were performed on an ESCALAB 250Xi XPS spectrometer (Thermo Scientific, USA), using a monochromated Al Kα source and equipped with an Ar^+^ ion sputtering gun. All binding energies were calibrated using the C (1 s) carbon peak (284.8 eV). ESI–MS data were acquired on a Waters Q-TOF mass spectrometer equipped with a Z-spray source. The sample was dissolved in toluene (∼1 mg ml^−1^) and diluted 1:1 in dry ethanol (5 mM CsOAc). The sample was directly infused at 5 μl min^−1^. The source temperature was fixed at 70 °C. The spray voltage was set at 2.20 kV and the cone voltage was set at 60 V.

### Single-crystal growth and analysis

Black crystals were formed from a CH_2_Cl_2_/hexane solution of the nanoclusters at 4 °C after 5 days. The diffraction data for Au_38_(PET)_24_ were collected at 173 K on a Bruker APEX DUO X-ray diffractometer using Cu Kα radiation (*λ*=1.54184 Å).

### Theoretical methods

All calculations were performed using density functional theory with the pure functional Perdew-Burke-Ernzerhof^45^,^46^ and the all electron basis set 6–31 g (*d*, *p*) for H, S, pseudopotential basis set LANL2DZ for Au, as implemented in the Gaussian 09 program package^47^. Time-dependent density functional calculations^48^ were performed to reproduce the experimental ultraviolet–visible spectrum and –R group was replaced by –H to minimize computational work^31^. The Gaussian half-width at half-height of 0.15 eV in the Multiwfn software^49^ was used to simulate the ultraviolet–visible spectrum.

### General procedure for the catalyses

4-Nitrophenol (69.50 mg, 0.500 mmol), Au_38Q_, Au_25_ or Au_38T_ (0.100 mol%, not adsorbed on a support or calcined) and THF (5 ml) were mixed in a reaction tube at 0 °C. The mixture was stirred at this temperature for 5 min. NaBH_4_ (189.00 mg, 5.000 mmol) dissolved in 1.0 ml of H_2_O was added slowly to the mixture. After stirring at 0 °C for 30 min, a large amount of water was added to quench the reaction. The mixture was extracted with dichloromethane twice (2 × 10 ml) and then the organic layers were collected and concentrated. The reduction product (4-aminophenol) was purified by column chromatography on silica gel, with ethyl acetate and petroleum ether (ethyl acetate/petroleum ether=1/1) as the eluant.

### General procedure for the recovery of Au_38T_

When the reduction was completed, the reaction mixture was quenched with water. Au_38T_ and other organic compounds were extracted with dichloromethane. The extract was collected and concentrated. After the other organic compounds were isolated by column chromatography with ethyl acetate and petroleum ether, Au_38T_ was recovered using dichloromethane as the eluant. The dichloromethane was evaporated under reduced pressure and then Au_38T_ was re-used in the next cycle without further treatment.

## Additional information

**Accession codes:** The X-ray crystallographic coordinates for structures reported in this study (see Supplementary Table 2 and Supplementary Data 1) have been deposited at the Cambridge Crystallographic Data Centre (CCDC), under deposition number CCDC 1423153. These data can be obtained free of charge from The Cambridge Crystallographic Data Centre via www.ccdc.cam.ac.uk/data_request/cif.

**How to cite this article:** Tian, S. *et al*. Structural isomerism in gold nanoparticles revealed by X-ray crystallography. *Nat. Commun.* 6:8667 doi: 10.1038/ncomms9667 (2015).

## Supplementary Material

Supplementary Information Figures 1-6 and Supplementary Tables 1-2

Supplementary Data 1 Crystallographic information file for Au38T
