# Erratum: Structural isomerism in gold nanoparticles revealed by X-ray crystallography

**DOI:** 10.1038/ncomms10012

**Published:** 2015-11-17

**Authors:** Shubo Tian, Yi-Zhi Li, Man-Bo Li, Jinyun Yuan, Jinlong Yang, Zhikun Wu, Rongchao Jin

*Nature Communications* 6: Article number: 866710.1038/ncomms9667 (2015); Published: 10202015; Updated: 11172015.

The original version of this Article contained an error in the spelling of isomerism in the title of the paper. This has now been corrected in both the PDF and HTML versions of the Article.

