# Supplementary material for: Structural isomserism in gold nanoparticles revealed by X-ray crystallography
Source: Nat Commun. 2015 Oct 20;6:8667. doi: 10.1038/ncomms9667 (PMC4667693; doi:10.1038/ncomms9667)
Supplement: Supplementary Information — Figures 1-6 and Supplementary Tables 1-2 [file ncomms9667-s1.pdf]

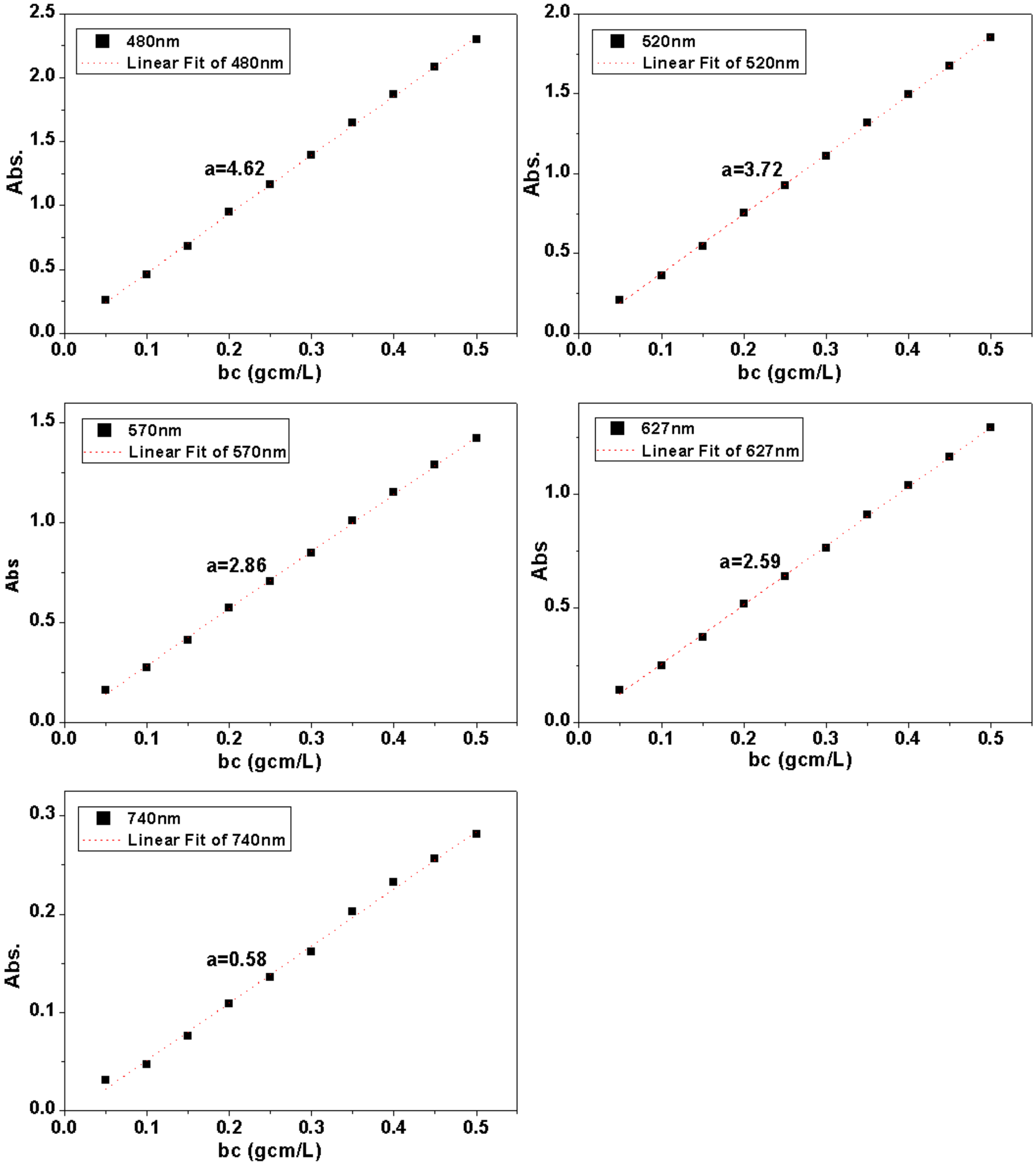

Supplementary Figure 1. The extinction coefficient of Au_{38Q} at various wavelengths.

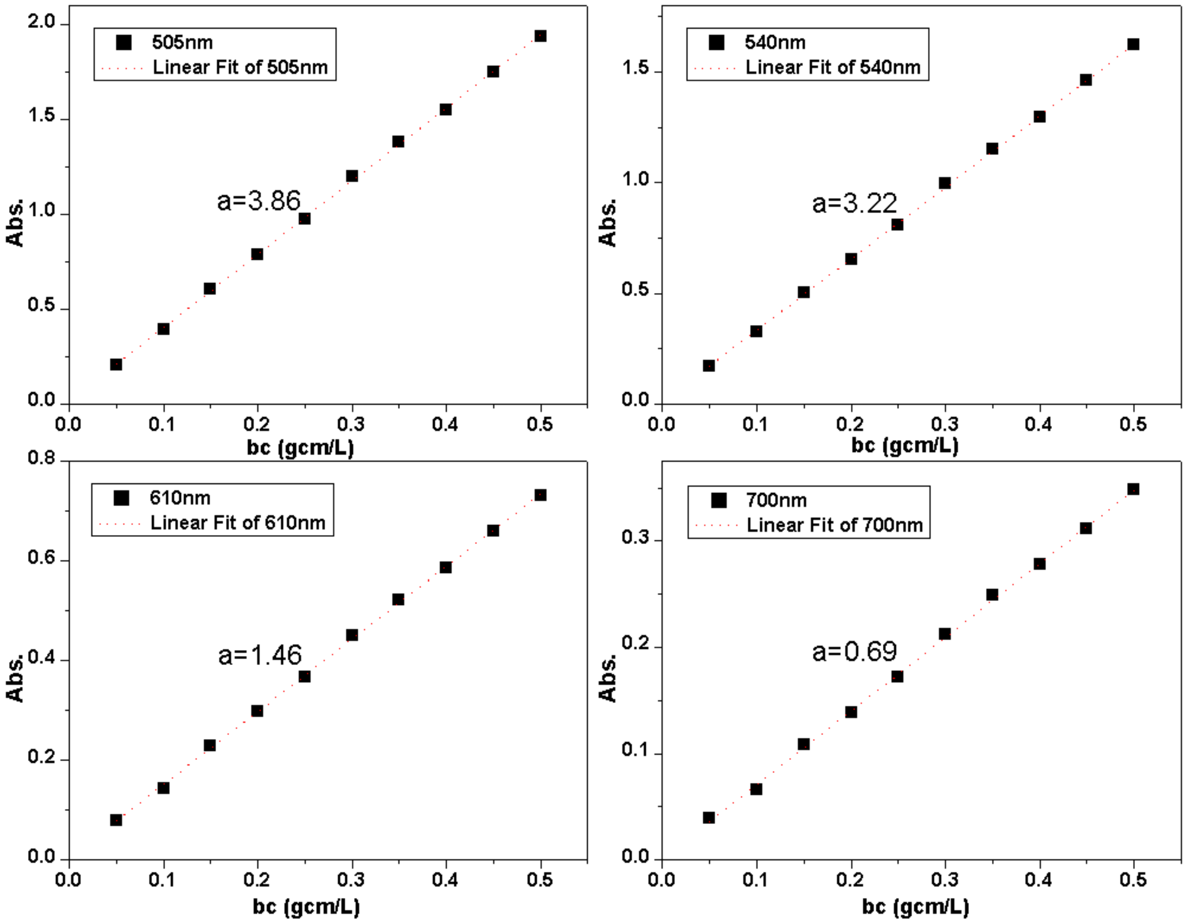

Supplementary Figure 2. The extinction coefficient of Au_{38T} at various wavelengths.

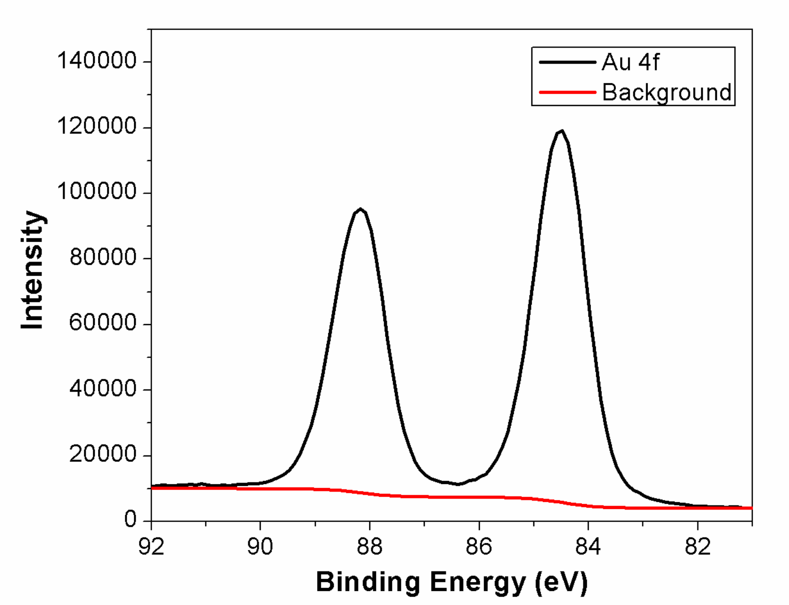

Supplementary Figure 3. Au4f XPS spectra of Au_{38T}.

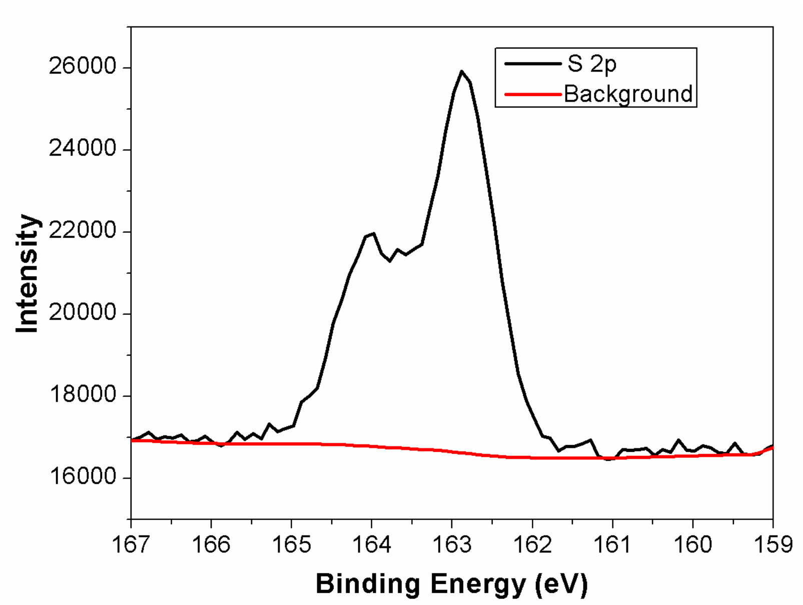

Supplementary Figure 4. S2p XPS spectra of Au_{38T}.

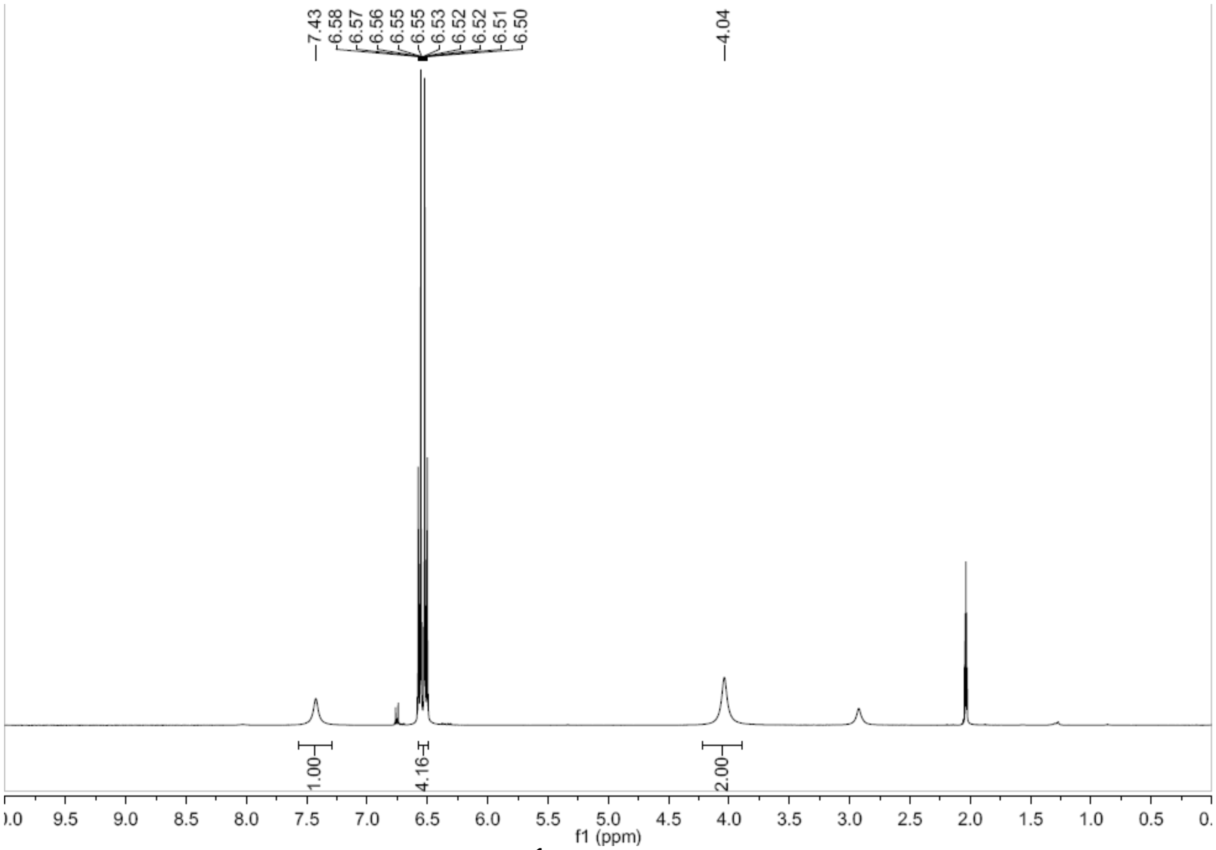

Supplementary Figure 5. ^{1}H NMR spectrum of 4-aminophenol.

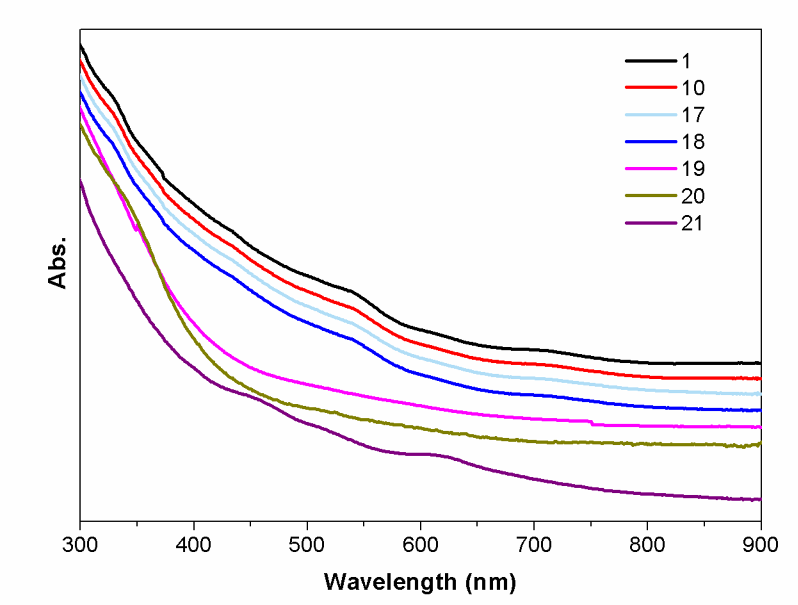

Supplementary Figure 6. The UV/Vis/NIR spectra of the catalyst after variously numbered cycles.

Supplementary Table 1. Recycle experiment of Au_{38T} [a]

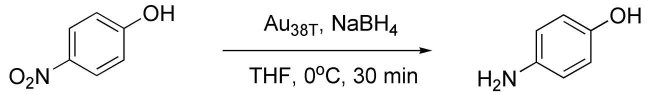

Entry	number of cycles	Yield[b] (%)
1	1	44
2	2	44
3	3	43
4	4	42
5	5	40
6	6	40
7	7	39
8	8	39
9	9	39
10	10	38
11	11	36
12	12	32
13	13	30
14	14	27
15	15	26
16	16	22
17	17	20
18	18	15
19	19	12
20	20	trace
21	21	0

[a] Reaction conditions: 4-nitrophenol (69.5 mg, 0.5 mmol), Au_{38T}(0.1 mol%), NaBH4 (190 mg, 5.0 mmol), THF (5.0 mL), [b] Isolated yield.

Supplementary Table 2. Crystal data and structure refinement for yy.

Identification code	yy	
Empirical formula	C192 H216 Au38 S24	
Formula weight	10777.87	
Temperature	173(2) K	
Wavelength	0.71073 Å	
Crystal system	Triclinic	
Space group	P -1	
Unit cell dimensions	a = 21.974(3) Å	a= 84.189(2)°.
	b = 22.685(3) Å	b= 79.598(3)°.
	c = 24.846(2) Å	g = 69.834(3)°.
Volume	11425(2) $Å^3$	
Z	2	
Density (calculated)	3.133 Mg/m^3	
Absorption coefficient	24.545 mm^{-1}	
F(000)	9508	
Crystal size	0.34 x 0.24 x 0.06 mm^3	
Theta range for data collection	0.83 to 27.00°	
Index ranges	-24<=h<=28, -28<=k<=28, -31<=l<=31	
Reflections collected	78414	
Independent reflections	48602 [R(int) = 0.0326]	
Completeness to theta = 27°	97.5 %	
Absorption correction	Semi-empirical from equivalents	
Refinement method	Full-matrix least-squares on F2	
Data / restraints / parameters	48602 / 0 / 1999	
Goodness-of-fit on F2	1.045	
Final R indices [I>2sigma(I)]	R1 = 0.0349, wR2 = 0.0961	
R indices (all data)	R1 = 0.0546, wR2 = 0.1064	
Largest diff. peak and hole	2.577 and -3.790 e.$Å^{-3}$	
